# The Effective Control of Hyperuricemia in Cancer Patients: A New Recombinant Conjugated Variant of Urate Oxidase

**DOI:** 10.31557/APJCP.2021.22.2.627

**Published:** 2021-02

**Authors:** Abbas Najjari, Hamid Shahbazmohammadi, Eskandar Omidinia, Abolfazl Movafagh

**Affiliations:** 1 *Enzyme Technology Laboratory, Department of Biochemistry, Genetic and Metabolism Research Group,Pasteur Institute of Iran, Tehran, Iran. *; 2 *Department Medical Genetic, School of Medicine, Shahid Beheshti University of Medical Sciences, Tehran Iran. *

**Keywords:** Cancer, hyperuricemia, PASylation technology, urate oxidase (UOX)

## Abstract

**Objective:**

Management of hyperuricemia is crucial to controlling tumor lysis syndrome (TLS) during cancer therapy. Urate oxidase (UOX) that catalyzes the enzymatic oxidation of uric acid into allantoin, is effective in lowering plasma uric acid levels and controlling hyperuricemia. Recently, we developed a new recombinant conjugate variant of UOX therapeutic drug using PASylation technology. This study was designed to evaluate the stability, plasma half-life and immunogencity of PASylated UOX.

**Methods:**

A recombinant variant of PASylated UOX from the *Aspergillus flavus *was manufactured using bioinformatics and experimental techniques. Ex vivo evaluation of stability of PASylated UOX was done in 50% human serum. For half-life test, recombinant PASylated UOX and rasburicase were administered at 1.5 mg/kg to 10 rats in two different groups and samples were collected after injection Production of antibodies against PASylated drug was also assayed.

**Results:**

Residual activity of PASylated UOX in 50% human serum was higher than rasburicase and native UOX. Stability of PASylated UOX at 25°C and 37°C was also higher than rasburicase and native UOX. The PASylated half-life was ~32.1 hours, whereas half-life for rasburicase and native UOX was ~25.1 and ~22.8 hours, respectively. In immunogenicity examination, there is 33% and 36% decrease in the absorbance of native UOX and rasburicase, respectively when compared with that of PASylated UOX.

**Conclusion:**

Our data confirmed the efficacy and stability of PASylated UOX in comparison to the rasburicase. In summary, the results indicated that PASylated UOX drug is effective at lowering plasma uric acid levels with prolonged plasma half-life and decreased cost.

## Introduction

Tumor lysis syndrome (TLS) is a chemotherapy-related side effect that destroys normal homeostatic mechanisms. TLS usually occurs during chemotherapy that results in hyperuricemia, hyperphosphatemia, and hypocalcemia (Hummel et al., 2005; Garay et al., 2012). On the other hand, managing hyperuricemia is crucial to controlling TLS. The most common method of hyperuricemia management to prevent acute renal failure is the use of allopurinol. The limitations of allopurinol are slow onset of action, and insufficient efficacy in high-risk patients (Cairo and Bishop, 2004). Conversion of uric acid to allantoin, a more water-soluble compound, by urate oxidase (UOX, oxygen oxidoreductase, E.C.1.7.3.3) is an alternative approach to inhibit uric acid formation (Keilin, 1959). UOX has been shown to be a more effective drug than allopurinol for the prophylaxis and treatment of TLS. This enzyme exists in many mammalians but it is inactive in humans owing to nonsense mutations in the coding region (Hayashi et al., 2000; Chen et al., 2008). An advantage of UOX is its ability to normalize uric acid levels by depleting extra uric acid. Recombinant UOX (Rasburicase) is currently approved for the treatment of acute hyperuricemia in patients receiving chemotherapy (Bosly et al., 2003; Cammalleri and Malaguarnera, 2007). This homotetrameric enzyme contains 301 residues which is expressed in an engineered *Saccharomyces cerevisiae *(Uegn, 2005). Rasburicase has been approved under the name of Fasturtec in Europe and under the name of Elitek by the USA Food and Drug Administration (FDA) for treatment of hyperuricemia during chemotherapy (Pession et al., 2008; Kennedy et al., 2011). Although, rasburicase indicates considerable reduction in serum uric acid concentration versus allopurinol, there are some issues that influence its efficacy (Renyl et al., 2007). Immunogenicity, short half-life and cost make rasburicase an unlikely choice for treatment of chronic gout and TLS. These problems can be resolved by masking the enzyme surface using soluble polymers, a process called bioconjugation (Punnappuzha et al., 2014). In other words, polyethylene glycol (PEG)ylation technique has been proposed to prolong half-life and reduce immunogenicity (Sundy et al., 2007). PEGloticase (KRYSTEXXA), a PEGylated UOX with a prolonged half-life, and reduced immunogenicity has been approved by FDA (Sherman et al., 2008; Nyborg1 et al., 2016). However, expensive purification of PEGylation, the decrease in activity of target enzyme, and non-degradability of PEG have somewhat limited this strategy. 

The biological alternative to PEGylation is PASylation technology, which the sequences comprising three amino acids including proline (Pro, P), alanine (Ala, A) and serine (Ser, S), are used for genetic conjugation to target protein (Binder and Skerra, 2017). PAS sequences have high stability and solubility. In contrast, they show no immunogenicity and interference with the pharmacological activity of other drugs. Furthermore, PAS sequences provide random coil structures formation, which results in increment the hydrodynamic volume of conjugate (Schlapschy et al., 2013). PASylation has successfully been applied to increase the half-life of conjugated therapeutic drugs, i.e., hormones, cytokines, antibody fragments, and enzymes. Recently, we developed a new conjugate variant of UOX enzyme through PASylation technology (Najjari et al., 2020). This communication was designed to evaluate the efficacy and plasma half-life of PASylated UOX.

**Figure 1:**
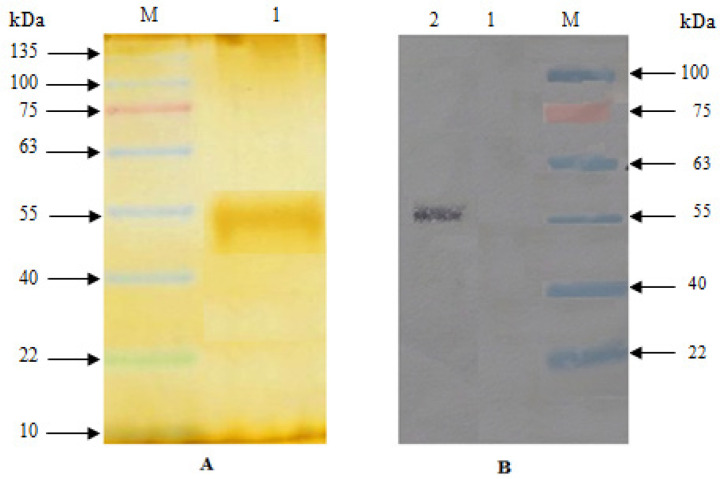
Production Analysis of Recombinant PASylated UOX by SDS-PAGE and Western Blotting Methods. (A) SDS-PAGE. Lane M: protein marker; lane 1: purified PASylated UOX. (B) Western blot. Lane M: protein marker; lane 1: Non-transformed E. coli BL21 (DE3); lane 2: purified PASylated UOX

**Figure 2:**
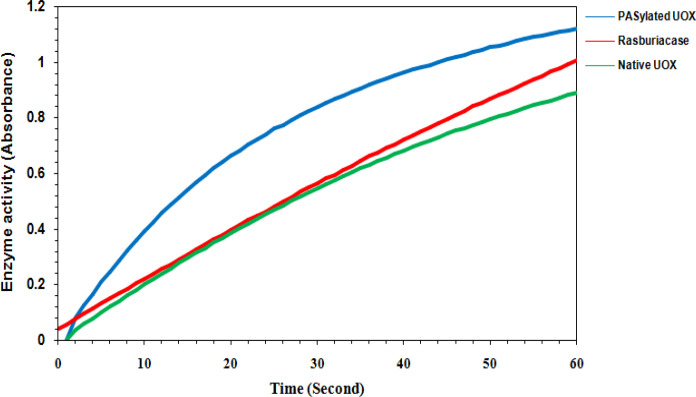
Enzymatic Activity Comparison of Native UOX, PASylated UOX and Rasburicase in Human Serum. The rate of uric acid oxidation was calculated based on a linear increase in absorbance

**Figure 3:**
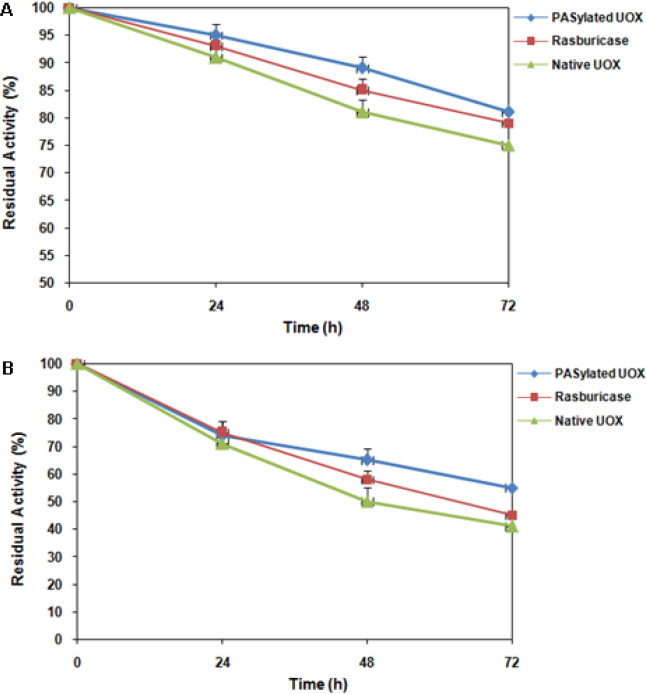
Stability of Native UOX, PASylated UOX and Rasburicase at 25°C (A) and 37°C (B).

**Figure 4:**
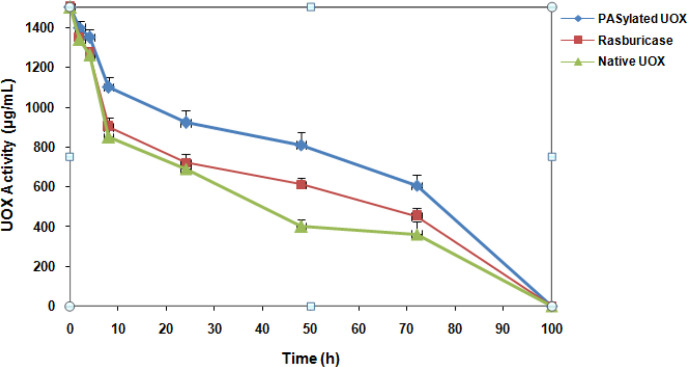
*In vivo* Plasma Half-Life Comparison of Native UOX, PASylated UOX and Rasburicase

**Figure 5:**
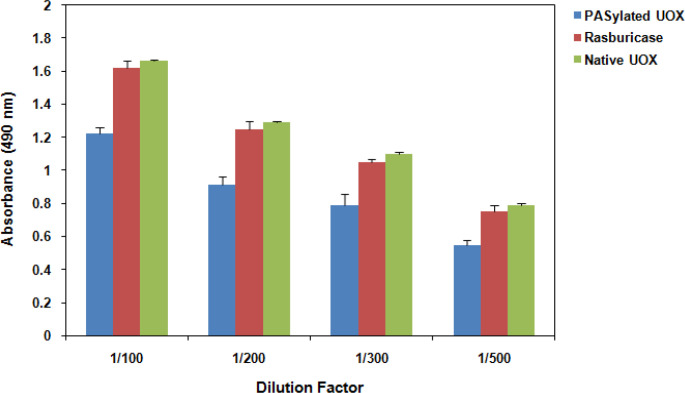
Antibody Affinity Analysis of Native UOX, PASylated UOX and Rasburicase Subjected to ELISA Method

## Materials and Methods

*Design, expression, purification and activity assay of native UOX and PASylated-UOX*

The synthetic coding DNA sequences of native UOX and PASylated UOX were cloned into pET-42a (+) using the NdeI and XhoI restriction enzymes. The constructs were introduced into *Escherichia coli *BL21 (DE3) competent cells. Then, transformed cells were cultured in Luria-Bertani (LB) containing kanamycin (40 µg/ml) at 37ºC, and 180 rpm to reach OD600=0.6-0.8, and induced by adding 14 g/L lactose at 37°C and 250 rpm for 5 h. The cells were harvested by centrifugation at 4°C and 12,000 rpm for 20 min, and stored at -20°C for further use. Harvested cells were re-suspended in lysis buffer (50 mM Na2HPO4, 300 mM NaCl, 0.1 mM EDTA, 1% glycerol, pH 8.0), and sonicated for 10 min (15 s on and 10 s off). The supernatants were obtained by centrifugation (12,000 rpm for 40 min at 4°C), and used for enzyme activity assay experiments (Shahbazmohammadi and Omidinia, 2017). Native UOX and PASylated UOX were purified from clarified lysate using Ni-NTA affinity columns. Purified enzymes were analyzed by SDS-PAGE. 

*Ex vivo evaluation of stability of PASylated UOX*

PASylated UOX was incubated in 50% human serum for 0 to 24 hours at 37°C and assayed for activity. Assay reaction was quenched at various time points using 50% Trichloroacetic acid which precipitates protein material but not uric acid (Xianyu et al., 2013). To study the temperature stability, PASylated UOX was incubated at two different temperatures such as 37°C and 25°C in 20 mM sodium tetraborate buffer (pH 9.0) and residual activity was measured at 0, 24, 48 and 72 h. Rasburicase (Fasturtec, Italy) and native UOX were used as controls and there results were compared with PASylated UOX.

*Sterility and pyrogen tests*

For pyrogen test, 0.1 mg of native UOX, PASylated UOX and rasburicase were injected to rabbits. Each group consisted of three rabbits. The baseline rectal temperature was recorded every 15 minutes for one hour before the injection. Temperatures were recorded every 15 minutes for three hours after the injection. The test indicates the presence of a pyrogen when one of the three rabbits shows an increase in body temperature higher than 0.5°C or when the sum of the maximum temperature rises in the three rabbits exceeds 1.2°C. For sterility test, native UOX, PASylated UOX and rasburicase were cultured in nutrient agar, blood agar, and Macconkey agar mediums. Media were incubated in aerobic and anaero¬bic conditions and observed after incubation at 37°C for 24 to 48 hours. 

*Plasma half-life study of PASylated UOX*

Wild type 9 week old rats were purchased from Pasteur Institute of Iran, Karaj, Iran. Three rats in each group were dosed at 1.5 mg/kg of native UOX, PASylated UOX or rasburicase and three samples were collected (Day -1), 2, 4, 8, 24, 48, 72, 96 and 100 hours post injection per rat. Whole blood was collected in serum separator tubes. Serum samples were diluted into saline phosphate buffer (PBS, pH=7.4) and activity assay was performed as described above.

*Immunogenicity analysis of PASylated UOX*

For immunogenicity assay, 15 rabbits (five per group) were randomly assigned to the native UOX, PASylated UOX and rasburicase groups. Each compound of UOX was administered through subcutaneous injection once weekly on days 0, 7, 14, 21, and 28 (designated as D0, D7, D14, D21, and D28). All serum samples were analyzed for detection of anti-UOX antibodies by ELISA method. Each well of ELISA plate was coated with 100 μL (4 μg/mL) of native UOX, PASylated UOX or rasburicase and incubated overnight at 4°C in sodium carbonate buffer (pH 9.6). After three washes with PBST, the plate was blocked using 300 μL PBS with 1% BSA for 1 h at 37°C. Blocking solution was then removed and all wells were washed three times in PBST. Serum samples were serially diluted by 1% BSA/PBS. After incubation at 37°C for 1 h and removal of samples, wells were washed three times in PBST and incubated with a 1:4,000 dilution of HRP-conjugated goat anti-rabbit IgG secondary antibodies at 37°C for 1 h. After washing, 100 μL of tetramethylbenzidine (TMB) substrate was added and incubated for 15 min at room temperature. Following addition of 50 μL of 2 N H_2_SO_4_, absorbance was measured at 450 nm. Blank serum sample was used as negative control. 

*Statistical analysis*

Each value represents the mean ± standard deviation (SD). Significant differences were determined using analysis of variance (ANOVA) to compare groups. Differences with p values below 0.05 were considered significant. Statistics were performed using GraphPad Prism 6 (GraphPad Software, La Jolla California USA).

## Results

*Analysis of produced PASylated UOX*

Analysis of PASylated UOX by SDS-PAGE and western blot confirmed that the enzyme of interest was uniformly conjugated with PAS sequences (Figure 1). As observed, PASylated UOX indicated a band at 55.0 kDa, which corresponds to the attachment of PAS sequences to one single subunit. 

*Ex vivo evaluation of stability*

The activity of native UOX, PASylated UOX and rasburicase was measured in 50% human serum (whole blood contains ~50% serum) at 37°C for 24 hours to mimic the complex in vivo matrix environment and temperature. Figure 2 shows comparable activity of native UOX, PASylated UOX and rasburicase in 50% human serum at 37°C. As can be found, residual activity of PASylated UOX was higher than rasburicase and native UOX. Stability of native UOX, PASylated UOX and rasburicase at two different temperatures for 72 hours was indicated in Figure 3. The achieved data showed that the stability of PASylated UOX at 25°C (Figure 3A) and 37°C (Figure 3B) was higher than rasburicase and native UOX.

*Half-life study of PASylated UOX*

The pharmacokinetic of PASylated UOX was investigated in rats. Rats were dosed at 1.5 mg/kg and serum samples were collected at various times and analyzed for UOX activity. Figure 4 demonstrates that PASylated UOX have substantially longer half-lives than the rasburicase and native UOX. The PASylated half-life was ~32.1 hours, whereas half-life for rasburicase and native UOX was ~25.1 and ~22.8 hours, respectively. 

*Immunogenicity examination of PASylated UOX*

Native UOX, PASylated UOX and rasburicase were coated on to the ELISA plates and the ability of anti-UOX antibody to bind them was compared as given in Figure 5. There is 33 % and 36 % decrease in the absorbance of native UOX and rasburicase, respectively when compared with that of PASylated UOX

## Discussion

Hyperuricemia results in gouty arthritis and chronic renal disease. Severe hyperuricemia occurs during TLS in cancer chemotherapy which may lead to metabolic disorders and death. Hyperuricemia do not develop in most mammals because they have the gene which encodes UOX which metabolizes the less soluble uric acid into a much more soluble metabolite. However, in humans, UOX gene has evolved to contain a nonsense codon which leads to a complete loss of enzyme activity. For this reason, scientists administrate UOX enzyme as a drug to patients. Although this treatment reduces plasma uric acid level, however, this therapeutic enzyme is costly, immunogenic and cause allergic reactions and anaphylaxis. Formulation of therapeutic proteins with PEG has been shown to reduce their anti-genicity and extend their circulating half-life. However, expensive purification of PEGylation and non-degradability of PEG have limited this strategy. PASylation technology has successfully been applied to increase the half-life of conjugated therapeutic drugs, i.e., hormones, cytokines, antibody fragments, and enzymes. Recently, we engineered A. falvus UOX enzyme through PASylation technology with improved properties including homogeneous product, biodegradability and cheap manufacturing. The A. flavus UOX was used to generate recombinant PASylated UOX that was purified from E. coli in soluble form (Najjari et al., 2020). The enzyme was engineered to increase plasma half-life and reduce immunogenicity. This work focuses on investigating the efficacy and plasma half-life of UOX-PAS100. The activity of native UOX, PASylated UOX and rasburicase was measured in 50% human serum at 25°C and 37°C. Residual activity of PASylated UOX was higher than rasburicase and native UOX. Stability of PASylated UOX was also higher than rasburicase and native UOX. The PASylated half-life was ~32.1 hours, whereas half-life for rasburicase and native UOX was ~25.1 and ~22.8 hours, respectively. Rasburicase has a high rate of clinical immunogenicity (Sundy et al., 2007). However, there is 33 % and 36 % decrease in the absorbance of native UOX and rasburicase, respectively when compared with that of PASylated UOX. Generally, immunogenicity has not been observed for PASylated therapeutics (Binder and Skerra, 2017). Our data suggest that the protein is low risk when it comes to clinical immunogenicity, but this remains to be tested.

Based on the in vivo activity, and immunogenicity studies, PASylated UOX described herein has the potential to provide a cost-benefit and effective therapy to patients. Animal models are not predictive of clinical immunogenicity so human clinical trials will be required to further understand immunogenicity, dose level and frequency. Collectively, comparing the data indicates that the modification of UOX with biodegradable and non-immunogenic PAS has significantly improved immunological properties of UOX. 
